# Comorbidity Assessment in Skin Cancer Patients: A Pilot Study Comparing Medical Interview with a Patient-Reported Questionnaire

**DOI:** 10.1155/2015/953479

**Published:** 2015-06-09

**Authors:** Erica H. Lee, Rajiv I. Nijhawan, Kishwer S. Nehal, Stephen W. Dusza, Amanda Levine, Amanda Hill, Christopher A. Barker

**Affiliations:** ^1^Department of Medicine, Dermatology Service, Memorial Sloan Kettering Cancer Center, New York, NY 10022, USA; ^2^Department of Dermatology, University of Texas Southwestern Medical Center, Dallas, TX 75390, USA; ^3^Department of Radiation Oncology, Memorial Sloan Kettering Cancer Center, New York, NY 10065, USA

## Abstract

*Background.* Comorbidities are conditions that occur simultaneously but independently of another disorder. Among skin cancer patients, comorbidities are common and may influence management.* Objective.* We compared comorbidity assessment by traditional medical interview (MI) and by standardized patient-reported questionnaire based on the Adult Comorbidity Evaluation-27 (ACE-27).* Methods.* Between September 2011 and October 2013, skin cancer patients underwent prospective comorbidity assessment by a Mohs surgeon (MI) and a radiation oncologist (using a standardized patient-reported questionnaire based on the ACE-27, the PRACE-27). Comorbidities were identified and graded according to the ACE-27 and compared for agreement.* Results.* Forty-four patients were evaluated. MI and PRACE-27 identified comorbidities in 79.5% and 88.6% (*p* = 0.12) of patients, respectively. Among 27 comorbid ailments, the MI identified 9.9% as being present, while the PRACE-27 identified 12.5%. When there were discordant observations, PRACE-27 was more likely than MI to identify the comorbidity (OR = 5.4, 95% CI = 2.4–14.4, *p* < 0.001). Overall comorbidity scores were moderate or severe in 43.2% (MI) versus 59.1% (PRACE-27) (*p* = 0.016).* Limitations.* Small sample size from a single institution.* Conclusion.* Comorbidities are common in skin cancer patients, and a standardized questionnaire may better identify and grade them. More accurate comorbidity assessments may help guide skin cancer management.

## 1. Background

Skin cancer is the most common malignancy worldwide. Management recommendations rely primarily on characteristics of the cancer and patient. One important patient characteristic is comorbidity or the simultaneous presence of a medical condition independent of skin cancer. Comorbidities have been shown to predict outcomes such as infection rates, quality of life, life expectancy, and complications in cancer management, with a trend indicating inferior outcomes in individuals with additional comorbidities [1–5]. Comorbidity severity has been shown to guide treatment selection in a range of conditions from oral cancer to end-stage renal disease [6–9].

A method for assessing comorbidity in a standardized fashion could be valuable in the management of skin cancer patients; however, there are no specific tools for comorbidity evaluation for this population. The Charlson Comorbidity Index (CCI), a validated measure to predict survival, is frequently cited in publications [10]. A recent study evaluated comorbidities in patients ≥90 years old treated with Mohs micrographic surgery (MMS) and found no significant difference in survival among patients with and without comorbidities [11]. On the contrary, another study assessed patients ≥80 years old who underwent dermatologic surgery and found that comorbidities were associated with increased mortality [12]. Similarly, a study of those 90–99 years of age treated with Mohs surgery reported that patients with CCI scores of zero (no comorbidities) had longer survival compared to patients with CCI of three (advanced comorbidities) [13].

In addition to the CCI, there are several validated tools used to assess comorbidity such as the Kaplan-Feinstein Index, Adult Comorbidity Evaluation-27 (ACE-27), and the Elixhauser Comorbidity Measure [14–18]. In the general cancer population, the ACE-27 is a validated instrument used to identify and grade 27 comorbidities among nine organ systems plus substance abuse, obesity, and malignancy [14]. It was developed through modification of the Kaplan-Feinstein Comorbidity Index and validated in 19,268 cancer patients treated at Barnes-Jewish Hospital [5]. Studies have shown that the ACE-27 can detect comorbidities more often than the CCI [19–21] and also led to deviations from standard management in patients with comorbidities that were detected by the ACE-27 [9, 22].

In this pilot study, we sought to compare comorbidity assessments collected by the traditional medical interview (MI) in a dermatologic surgery practice and by a standardized patient-reported questionnaire based on the ACE-27 (referred to as PRACE-27 in this paper) ascertained by a radiation oncologist. We specifically used ACE-27 given its validation in a variety of cancer populations, its comprehensive approach, and its application using retrospective note review by cancer registrars [23, 24]. The objectives of this pilot study were to (1) assess the frequency, severity, and type of comorbidities among skin cancer patients presenting for outpatient management and (2) identify discrepancies in comorbidity assessment between MI and PRACE-27.

## 2. Methods

IRB exemption was obtained at Memorial Sloan Kettering Cancer Center. All patients with a biopsy proven skin cancer evaluated by both a radiation oncologist (CB) and one of two dermatologic surgeons (KN, EL) within a six month period between September 2011 and October 2013 were identified. Comorbidities were ascertained in a prospective manner using one of two techniques. The Mohs surgeons characterized comorbidities through a traditional medical interview (MI). The radiation oncologist performed comorbidity assessment by a patient-reported questionnaire based on the ACE-27 (referred to as PRACE-27).

Medical records were retrospectively reviewed and comorbidities were identified and graded according to the ACE-27. Individual organ systems and diseases were assessed based on specific criteria outlined by the grading guidelines on the following scale: 0 = no disease, grade 1 = mild decompensation, grade 2 = moderate decompensation, and grade 3 = severe decompensation. Based on the grading of the individual organ systems, an overall comorbidity score was calculated on a scale from 0 (no comorbidity) to 3 (severe comorbidity).

### 2.1. Statistical Analysis

The distributions of assessments for the twenty-seven comorbidities were evaluated for MI and PRACE-27. Cross-classifications between MI and PRACE-27 were completed to assess comorbidity prevalence, percent agreement, kappa, and weighted kappa. The kappa (*κ*) value interpretations were graded as poor (*κ* < 0.2), fair (*κ* = 0.2–0.4), moderate (*κ* = 0.41–0.6), substantial (*κ* = 0.61–0.8), or excellent (*κ* = 0.81–1.0). McNemar's Chi-square and odds ratios with 95% confidence intervals were estimated for the paired observations. Trends in survival were also assessed. All statistical analyses were performed with Stata v.12.1, Stata Corporation, College Station, TX.

## 3. Results

Forty-four patients were studied. Patient demographic, cancer, and comorbidity data are listed in Table 1. Overall, 79.5% and 88.6% (*p* = 0.12) of patients were identified as having a comorbidity, according to the MI and PRACE-27, respectively. The most common comorbid ailments were hypertension, solid tumor, respiratory disease, and cardiac arrhythmia. Overall comorbidity scores were moderate or severe (scores of 2 or 3) in 43.2% (MI) versus 59.1% (PRACE-27) of patients, and this difference was statistically significant (*p* = 0.016; difference in paired proportions = 0.159 (95% CI: 0.028–0.29)).

As noted in Table 2, percent agreement and kappa scores for comorbidity identification in comorbid ailments between the MI and PRACE-27 were excellent (96%, range: 77.3–100%, kappa = 0.81). Among the comorbid ailments assessed by the ACE-27, 9.9% were identified using MI, while 12.5% were identified using the PRACE-27. When there were discordant observations, PRACE-27 was more likely than MI to identify the comorbidity (OR = 5.4, 95% CI = 2.4–14.4, *p* < 0.001). The respiratory system had the lowest percent agreement (94.7%), and, for this organ system, 10 patients were graded at a higher severity with the PRACE-27 compared to the MI.  Table 3 presents the percent agreement and kappa between MI and PRACE-27 assessments for data grouped by organ system.

The PRACE-27 identified 4 patients (9%) with one or more comorbidities (related to respiratory disease, obesity, and gastrointestinal disease) that the MI identified as having no comorbidity, compared to 3 patients (6.8%) identified by the MI (related to solid organ malignancy) that was missed by the PRACE-27 (*p* = 0.38). PRACE-27 identified a greater severity of comorbidity when both assessments agreed to its presence. PRACE-27 rated 8 patients with a higher severity than the MI, whereas MI did not increase the severity score for any patients compared to PRACE-27. While there were discrepancies in the comorbidity grading severity of individual organ systems, the agreement in the overall comorbidity score for the patients was high (91.7%, *p* < 0.001) (Table 4).

The median follow-up for the 44 patients was 1.9 years from the first comorbidity assessment to the most recent follow-up. There were 7 deaths of which 5 (71.4%) were graded by the PRACE-7 as severe (i.e., 3) and 3 (42.8%) were graded by the MI as severe. Across comorbidity groups, there was a trend for lower survival for higher comorbidities that was significant for both the PRACE-7 and the MI groups (*p* < 0.05). Two-year survival rates were estimated as 100%, 92%, 91%, and 68% for grades 0, 1, 2, and 3 using the PRACE-27. Two-year survival rates were 100%, 86%, 91%, and 57% for grades 0, 1, 2, and 3 using the MI. These estimations are relatively similar to previously published estimations using the ACE-27: grade 0 (83%), grade 1 (80%), grade 2 (72%), and grade 3 (66%) [5].

## 4. Comment

The National Comprehensive Cancer Network (NCCN) emphasizes the importance of collecting and measuring comorbidities in cancer patients to guide medical care and for research. For skin cancer patients, comorbidities are most commonly assessed using standard medical interview (MI) and rarely graded with a standardized approach. We sought to compare comorbidity collection between MI and a standardized patient-reported questionnaire based on the ACE-27 (PRACE-27).

Identification and agreement of comorbidities across 27 ailments as they relate to individual organ systems were high between the two data collection methods; however, PRACE-27 was more likely than MI to identify the existence of an ailment and determine that an ailment was of a greater severity. However, MI appeared to identify solid tumor malignancies more frequently, suggesting that additional attention to capture this ailment may be needed when using the PRACE-27. Nevertheless, the systematic collection of comorbidities with a questionnaire (e.g., PRACE-27) ensures that all organ systems are comprehensively queried. Severity grading may also provide a more accurate assessment of disease status, the overall picture of a patient's health, and potential survival status. Based on this pilot study, a standardized patient-reported questionnaire may better identify and grade individual comorbidities in skin cancer patients compared to standard MI. The concordance in the overall comorbidity score, which is most commonly used to predict outcomes, was also high, suggesting that the PRACE-27 is an accurate assessment of comorbidities, compared to the gold-standard MI [25].

Comorbidity assessment using ACE-27 has been reported to be predictive of treatment modifications. In a study of cancer patients evaluated by a radiation oncologist, a change in treatment plan was noted for patients with moderate or severe indices more often than those with none or mild indices, while age had no contribution to predicting treatment change [22]. Comorbidity grading may similarly guide multidisciplinary management in the skin cancer population. Importantly, in skin cancer populations, patients with less comorbidity have also been shown to have better skin-related quality of life after treatment of both non-melanoma skin cancer (NMSC) and localized melanoma [26, 27]. However, the data is limited in the skin cancer population and additional research is needed to assess whether comorbidities affect outcomes and if systematic assessment should be included in skin cancer management algorithms.

There are a few limitations to this study. First, the number of patients studied was small and the follow-up period was limited to only 2 years. However, the power of the analysis is derived from the large number of binary endpoints used in the comparative analyses. Moreover, this pilot study required cotemporaneous evaluation by a small number of staff from two clinical services, thus limiting the number of potential patients eligible for study. Second, a single institution setting may not be representative of the general skin cancer population. Nevertheless, a study goal was to compare two methods of data collection, and identifying patients evaluated at a single center facilitated this objective. Third, comorbidity data extraction used information from an outpatient clinical assessment and did not utilize review of all of a patient's medical records. While this limited amount of clinical data may have led to underestimation of a patient's comorbid conditions, because most patients with skin cancer receive care by outpatient specialists, this is representative of general clinical practice. Moreover, retrospective chart review has previously been shown to provide sufficient information for comorbidity scoring using ACE-27 with minimal limitations [23, 24]. Finally, some might suggest that the results of the present study cannot be generalized because of the inability to implement a patient-reported comorbidity assessment in clinical practice. Although there are 27 comorbidities to identify and grade in the PRACE-27, there is a quick learning curve and computing severity scores takes less than three minutes [22].

Comorbidity assessment will likely become an important factor in individualizing treatment for all malignant diseases including skin cancer. It is well accepted that a single treatment approach should not be applied to all patients with skin cancer [28], and thus grading comorbidity severity in patients may help guide management. This pilot study shows that PRACE-27 was more likely to identify an ailment than the MI and better identified the higher grades. The ACE-27 is a validated tool that may be more applicable in the skin cancer population compared to other tools such as the Charlson Comorbidity Index (CCI), whose applicability to skin cancer populations has been challenged [28]. Utilizing a patient-reported questionnaire like the PRACE-27 during the physician encounter may ensure that important components of the relevant medical history are not omitted. Further research is needed to better delineate the role of comorbidity assessment in the skin cancer population.

## Figures and Tables

**Table 1:** Patient demographic data, lesion information, and comorbidity data.

Demographic data								
Average age in years (median; range)	73.5 (76; 25–94)							
Female	22 (50.0%)							
Male	22 (50.0%)							

Diagnoses								

Basal cell carcinoma	22 (50.0%)							
Squamous cell carcinoma	12 (27.3%)							
Melanoma	7 (15.9%)							
Dermatofibrosarcoma protuberans	1 (2.3%)							
Merkel cell carcinoma	1 (2.3%)							
Microcystic adnexal carcinoma	1 (2.3%)							

Comorbidity data MI versus PRACE-27								

	MI				PRACE-27			
	0	1	2	3	0	1	2	3

Comorbid ailment								
Myocardial infarction	43	1	—	—	40	2	2	—
Angina/coronary artery disease	38	6	—	—	37	5	2	—
Congestive heart failure	44	—	—	—	42	—	2	—
Arrhythmias	36	—	8	—	35	1	8	—
Hypertension	22	22	—	—	19	20	5	—
Venous disease	40	2	1	1	40	1	2	1
Peripheral arterial disease	44	—	—	—	42	—	2	—
Respiratory system	42	1	1	—	34	6	2	2
Hepatic	44	—	—	—	43	—	—	1
Stomach/intestine	44	—	—	—	44	—	—	—
Pancreas	44	—	—	—	44	—	—	—
End-stage renal disease	44	—	—	—	44	—	—	—
Diabetes mellitus	39	3	2	—	39	2	1	2
Stroke	39	3	2	—	37	6	1	—
Dementia	41	2	—	1	41	1	1	1
Paralysis	43	1	—	—	41	2	—	1
Neuromuscular	41	2	—	1	40	2	—	2
Psychiatric	42	2	—	—	42	1	1	—
Rheumatologic	44	—	—	—	43	1	—	—
AIDS	44	—	—	—	44	—	—	—
Solid tumor including melanoma	28	9	6	1	31	7	4	2
Leukemia and myeloma	42	1	1	—	42	1	1	—
Lymphoma	41	3	—	—	41	2	—	1
Alcohol	44	—	—	—	44	—	—	—
Illicit drugs	44	—	—	—	44	—	—	—
Obesity	44	—	—	—	41	—	3	—
Overall comorbidity score	9	16	12	7	5	13	13	13

The comorbidity data section compares the number of patients identified as having a comorbid ailment between the MI and PRACE-27. Individual organ systems and diseases were assessed based on specific criteria outlined by the grading guidelines on the following scale: 0 = no disease, grade 1 = mild decompensation, grade 2 = moderate decompensation, and grade 3 = severe decompensation. Based on the grading of these individual organ systems, an overall comorbidity score was calculated on a scale from 0 (no comorbidity) to 3 (severe comorbidity).

**Table 2:** Comorbid ailments captured and missed by medical interview (MI) and PRACE-27 in individual organ systems.

	Medical interview		Total
	Present	Absent	
PRACE-27			
Present	111	**38**	149
Absent	**7**	1032	1039
Total	118	1070	1188

There are a total of 1,188 possibilities for comorbid ailments to be identified among the forty-four patients in this study since each patient can potentially have twenty-seven comorbidities (44 × 27 = 1,188). Both MI and PRACE-27 agreed upon 111 comorbidities being present and 1032 being absent. However, PRACE-27 identified 38 comorbidities that were deemed as being absent by MI while MI identified 7 comorbidities that were deemed absent by PRACE-27 [OR = 5.4, 95% CI: 2.4–14.4, *p* < 0.001].

**Table 3:** Percent agreement and kappa between medical interview (MI) and PRACE-27 assessments for data grouped by disease system and overall comorbidity scores.

Organ system	Percent agreement	Kappa	*p* value
Cardiovascular	98.7	0.80	<0.001
Respiratory	94.7	0.40	<0.001
Gastrointestinal	99.2	—	—
Renal	100	—	—
Endocrine	98.7	0.84	<0.001
Neurologic	98.8	0.77	<0.001
Psychiatric	96.0	−0.04	0.62
Rheumatologic	97.7	—	—
Immunological	100	—	—
Malignancy	98.7	0.84	<0.001
Substance abuse	100	—	—
Obesity	100	—	—

**Table 4:** Percent agreement and kappa between medical interview (MI) and PRACE-27 assessments for overall comorbidity scores.

PRACE-27								
	Medical interview					Percent agreement	Kappa	*p* value
*Overall comorbidity score *	0	1	2	3	Total # patients	91.7	0.64	<0.001
0	5^*∗*^	0	0	0	5			
1	2^*∗∗*^	11^*∗*^	0	0	13			
2	1^*∗∗*^	2^*∗∗*^	9^*∗*^	1^*∗∗∗*^	13			
3	1^*∗∗*^	3^*∗∗*^	3^*∗∗*^	6^*∗*^	13			
Total # patients	9	16	12	7	44			

PRACE-27 and MI had identical overall comorbidity scores for 31/44 patients (marked with *∗*). There were 12 patients for whom PRACE-27 had a higher overall comorbidity score (marked with *∗∗*) compared to MI and 1 patient (marked with *∗∗∗*) for whom MI had a higher overall comorbidity score compared to PRACE-27.
